# Thermal Conductivity of VO_2_ Nanowires at Metal-Insulator Transition Temperature

**DOI:** 10.3390/nano11092428

**Published:** 2021-09-17

**Authors:** Da Li, Qilang Wang, Xiangfan Xu

**Affiliations:** Center for Phononics and Thermal Energy Science, China-EU Joint Center for Nanophononics, School of Physics Science and Engineering, Tongji University, Shanghai 200092, China; lida@tongji.edu.cn (D.L.); 1153570@tongji.edu.cn (Q.W.)

**Keywords:** thermal conductivity, size-dependent, metal−insulator transition, vanadium dioxide

## Abstract

Vanadium dioxide (VO_2_) nanowires endowed with a dramatic metal−insulator transition have attracted enormous attention. Here, the thermal conductance of VO_2_ nanowires with different sizes, measured using the thermal bridge method, is reported. A size-dependent thermal conductivity was observed where the thicker nanowire showed a higher thermal conductivity. Meanwhile, the thermal conductivity jump at metal−insulator transition temperature was measured to be much higher in the thicker samples. The dominant heat carriers were phonons both at the metallic and the insulating regimes in the measured samples, which may result from the coexistence of metal and insulator phases at high temperature. Our results provide a window into exploring the mechanism of the metal−insulator transition of VO_2_ nanowires.

## 1. Introduction

Metal-insulator transition (MIT) has long been a widely researched centerpiece in condensed matter physics, with a number of efforts focusing on potentially exploiting the resulting changes in the functional properties in novel electronics and phononics, as well as understanding the emergent phenomena. In the 1950s, Morin first noted that the electrical resistance in some transition metal oxides increases by several orders of magnitude when the temperature crosses the transition temperature [1]. In recent years, vanadium oxide compounds, as a kind of typical strongly correlated electron materials, have attracted considerable interest for MIT. Among them, VO_2_, whose phase transition temperature is close to room temperature, has great potential applications in Mott field-effect transistors [2,3], optical temperature sensors [4], ultrafast photoelectric switch materials [5] and thermochromic devices [6], resulting in tremendous studies and hot research topics ongoing.

VO_2_ undergoes a first-order MIT at around 68 °C from a high-temperature metallic (M) phase to a low-temperature insulating (I) phase. Accompanying the electronic transition is a structural phase transition from a high-temperature tetragonal structure to a low-temperature monoclinic structure, making the specimen spontaneously shrink by 1% along the tetragonal *c* axis [7,8]. Moreover, the magnetic susceptibility and optical constants of VO_2_ change dramatically in a narrow temperature interval of only a few degrees at around the MIT temperature [9]. Due to the novel physical properties of VO_2_, it has received more attention, especially concerning thermal transport. The thermal transport of VO_2_ nanowires has been studied mainly in two aspects. In one area, the interest is mainly focused on the change of thermal conductivity in VO_2_ nanowires across the MIT along with the underlying mechanism. For the other, the focus is on how to regulate the underlying mechanism. Oh et al. measured the thermal properties of VO_2_ thin film with a thickness of 90–440 nm by time-domain thermoreflectance across the MIT temperature and found that the thermal conductivity increased by as much as 60% in the metallic phase [10]. Xie et al. reported the realization of a solid-state thermal memory through an effective electrical control in a single-crystal VO_2_ nanobeam [11]. In addition, temperature [12], stress [13,14,15,16], doping [17,18], electric field [2,19] and hydrogenation [20,21] are important factors affecting the phase transition temperature, which pave the way for manipulating MIT and the consequent thermal properties. Despite these remarkable advantages, understanding of the thermal transport mechanism and thermal manipulation of VO_2_ is still deficient, considering the complex phase diagram as well as the complications arising from coexisting metal and insulator domains across the MIT. Besides, a crucial bottleneck in the thermal transport at nanoscale is the contact thermal resistance between the materials and the substrate, which may affect the thermal conductivity of the measured materials.

In this work, we experimentally measured the thermal conductivity of VO_2_ nanowires using the thermal bridge method and a size-dependent thermal conductivity was observed. Combining the electron-beam self-heating method, we managed to measure the contact thermal resistance between the samples and the suspended microdevice, which helped to measure the intrinsic thermal conductivity with extrinsic errors excluded. Moreover, we studied the thermal conductivity and electrical resistance of VO_2_ nanowires near the phase transition temperature, and found that the main heat carriers were phonons. These results are helpful for further understanding of MIT, as well as its promising device application in electronics.

## 2. Results and Discussion

**Figure 1:**
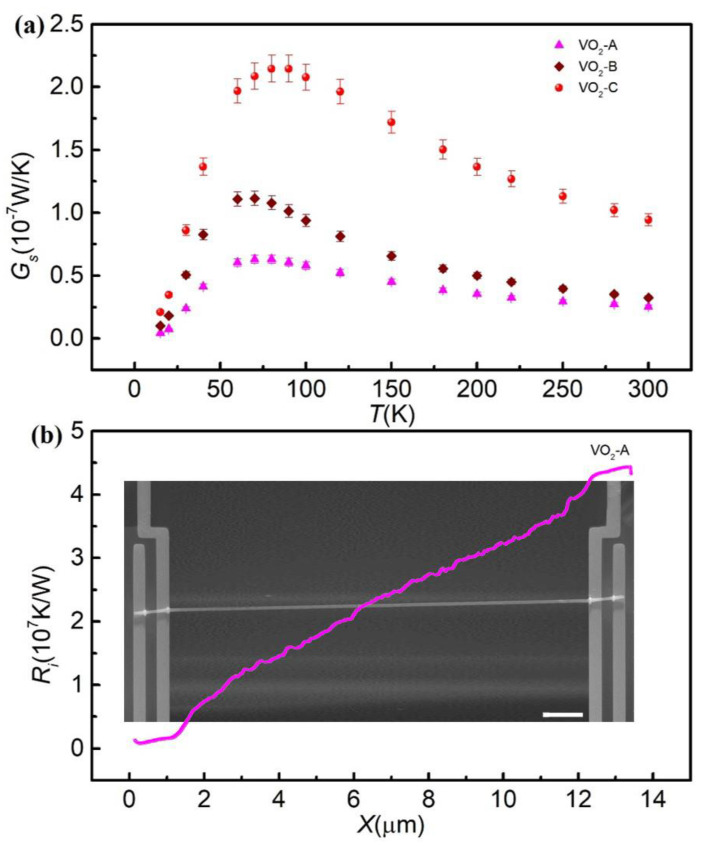
(**a**) Thermal conductance of the measured three VO_2_ nanowires as a function of temperature. (**b**) The intrinsic thermal resistance measured by the electron beam self-heating method varies with the scanning position of the electron beam for VO_2_-A. Inset: scanning electron microscopy images of the VO_2_ nanowires. The scale bar is 2 μm.

Monocrystalline VO_2_ nanowires were prepared via a variant of the vapor transport method reported previously [22]. Considering the effect of adhesion between VO_2_ nanowires and substrate, which may lead to the spontaneous formation of periodic, alternating M−I domain patterns along the VO_2_ nanowires’ length during MIT [23], single VO_2_ nanowire was transferred onto the suspended micro-electro-mechanical system (MEMS) device for thermal conductivity measurement using a tungsten needle in a micromanipulator (Imina Technologies Micromanipulation Platform). The MEMS device contains two thermally isolated thermometers for the measurement of thermal properties and four electrodes for the measurement of electrical properties and was suspended by the wet etching method. The inset in Figure 1b shows an exemplary scanning electron microscopy (SEM) image of the VO_2_ nanowire on the suspended MEMS device. To fix the VO_2_ nanowires, platinum (Pt) bars were deposited on the two ends of the VO_2_ nanowires by electron-beam induced deposition. The lengths of the VO_2_ nanowires changed from 11 µm to 20 µm, and their diameters ranged from 398 nm to 708 nm, labeled as VO_2_-A, VO_2_-B and VO_2_-C, respectively. The size parameters of the VO_2_ nanowires are displayed in Table 1.

Figure 1a shows the thermal conductance of VO_2_ nanowires versus temperature measured by the thermal bridge method. The overall measurement was performed in a high vacuum condition, better than 1 × 10^−5^ Pa. At a low temperature, the thermal conductance increased as the temperature increased, was mainly affected by phonon-boundary scattering and specific heat. The thermal conductance of VO_2_ nanowires reached a peak value of 2.14 × 10^−7^ W/K at *T* = 80 K for VO_2_-C. When the temperature was above 80 K, owing to phonon−phonon Umklapp scattering, the thermal conductance decreased as the temperature increased, and finally declined to 9.43 × 10^−8^ W/K at *T* = 300 K for VO_2_-C.

As the reciprocal of thermal conductance, the total thermal resistance (*R_s_*) obtained from the thermal bridge method contains two parts: the intrinsic thermal resistance (*R_i_*) of the suspended VO_2_ nanowires and the contact thermal resistance (*R_c_*) between the VO_2_ nanowires and the electrodes (Pt). The analysis of the thermal conductance would be trivial without considering the contact thermal resistance. To further determine the result of the contact thermal resistance measured in the thermal bridge method, we measured it at room temperature directly by the electron-beam self-heating method [24,25,26]. The electron-beam self-heating method was developed on the basis of the suspended thermal bridge method, where the temperature change is monitored by the resistance change of two suspended thermometers, *R_h_* and *R_s_*. Different from the suspended thermal bridge method, the electron beam serves as the heat source in the electron-beam self-heating method. In general, the electron-beam can cause great damage to organic matter, and has little impact on inorganic matter [27,28]. In this work, the incident energy of the electron beam applied to the sample was 15 keV, which has been proven safe for VO_2_ nanowires in previous work [28]. The electron beam slowly scanned the entire VO_2_ nanowire, and its temperature slowly increased due to the electron beam power absorption. Subsequently, the heat transferred through the nanowire to the suspended thermometers, raising their temperature Δ*T_L_* and Δ*T_R_*, respectively. The heat conduction equations can be obtained respectively:

(1) $$\frac{\Delta T_{i}\left(x\right)-\Delta T_{L}}{R_{i}\left(x\right)}=\frac{\Delta T_{L}}{R_{b}}$$

(2) $$\frac{\Delta T_{i}\left(x\right)-\Delta T_{R}}{R_{T}-R_{i}\left(x\right)}=\frac{\Delta T_{R}}{R_{b}}$$

where *R_i_*(*x*) is the total thermal resistance of VO_2_ nanowires from the start point to the focal point of the electron beam, Δ*T_i_*(*x*) is the temperature change at the focal point of the electron beam, *R_T_* and *R_b_* are the total thermal resistance of the VO_2_ nanowires and the six beams of the suspended device, respectively. According to these two formulas, *R_i_*(*x*) can be obtained:

(3) $$R_{i}\left(x\right)=R_{b}\left\{\frac{\alpha_{0}-\alpha_{i}\left(x\right)}{1+\alpha_{i}\left(x\right)}\right\}$$

where $\alpha_{0}=\Delta T_{L0}/\Delta T_{R0}$, $\alpha_{i}=\Delta T_{L}/\Delta T_{R}$. Δ*T_L_*_0_ and Δ*T_R_*_0_ are the temperature change of the two thermometers in the suspended thermal bridge method, respectively. By measuring the thermal resistance when an electron beam scans along the nanowire, the thermal conductivity of the VO_2_ nanowires can be calculated as:

(4) $$\kappa =\frac{1}{\left(\frac{dR_{i}}{dx}\right)\cdot A}$$

where *A* is the cross-sectional area of VO_2_ nanowires. Considering that the electron-beam self-heating method is carried out in SEM, it is usually applied to the measurement of thermal conductivity at room temperature.

Figure 1b shows the thermal resistance *R_i_* of VO_2_ nanowires measured by the electron beam self-heating method as a function of scanning length at room temperature. The total measured intrinsic thermal resistance of VO_2_ nanowires is 2.97 × 10^7^ K/W at room temperature for VO_2_-A. The contact thermal resistance is obtained by subtracting the intrinsic thermal resistance from the total thermal resistance measured from the thermal bridge method. The contact thermal resistance was calculated to be 9.8 × 10^6^ K/W for VO_2_-A at room temperature. The contact thermal resistance accounted for 21~25% of total thermal resistance, indicating that the contact thermal resistance did indeed influence the thermal conductance of the VO_2_ nanowires. At low temperatures, the contact thermal resistance increased with increasing temperature. However, at high temperature, the contact thermal resistance increased and finally closed to an asymptotic value. Therefore, it is safe to assume that the contact thermal resistance at a higher temperature is similar to that at 300 K.

**Figure 2:**
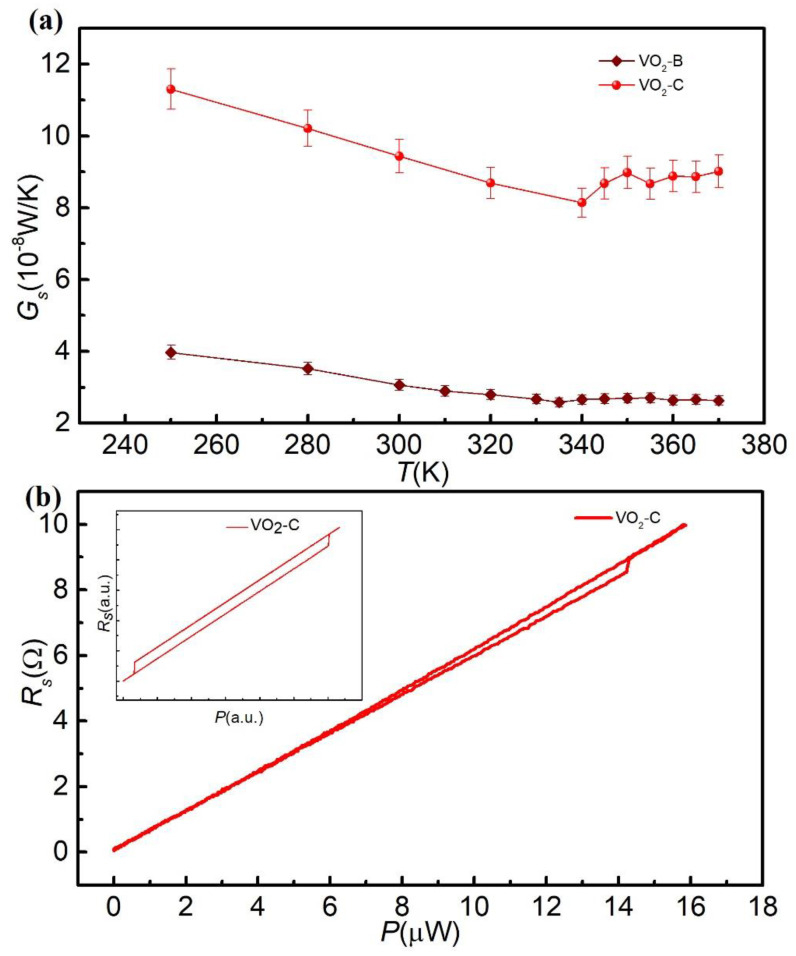
(**a**) The thermal conductance as a function of temperature for VO_2_-B and VO_2_-C, respectively. (**b**) The electrical resistance of the sensor as a function of the heating power using the thermal bridge method at *T* = 335 K. The inset shows the simulation results of electrical resistance of sensor as a function of the heating power in the thermal bridge method at *T* = 335 K.

The thermal conductance of VO_2_ nanowires above room temperature are plotted in Figure 2a. Clearly, the thermal conductance dependence on temperature revealed that the samples underwent MIT [12]. The thermal conductance decreased with the increasing temperature under the phase transition temperature. Above the phase transition temperature, the VO_2_ nanowires were transformed into the metal phase and the thermal conductance increased as the temperature increased. The *η* is the ratio of the thermal conductivity change to the thermal conductivity in the I phase during the MIT, defined as $\eta =(\kappa_{I}-\kappa_{M})/\kappa_{I}$, where *κ_M_* and *κ_I_* are the thermal conductivity in the M phase and I phase, respectively. The measured *η* is 4.12% in sample VO_2_–B. The *η* is smaller than that reported in VO_2_ films [10,12], which may be attributed to stronger boundary scattering. This result will be discussed in more detail later. The phase transition temperature is in the range of 335–340 K for bothVO_2_-B and VO_2_-C, lower than previous reports from electric measurement, probably due to the temperature rise during thermal measurement [29].

To understand more details of MIT, we modified the measurement method. The VO_2_ nanowires were fixed to the thermal bridge device, and the measurement temperature was set to be 335 K, slightly below MIT temperature. A DC current was applied to the heater, increasing from 0 to 150 µA, then gradually decreasing to 0. The temperature of the heater increased by nearly 27 K when the heating power reached the maximum of 15.89 μW. There was no doubt that the VO_2_ nanowires underwent MIT during the heating process since the resistance of the sensor had obviously jumped with the increase of heating power. As shown in Figure 2b, the resistance of the sensor jumped about 0.39 Ω with the increasing DC current at 335 K, which accounted for 3.95% of the total resistance variation. This behavior indicated great changes had taken place in the thermal conductance of the VO_2_ nanowires.

It is worth noting that the jump only occurred during the heating process, and the resistance displayed a linear behavior with the heating power during the cooling process. It is reasonable that a hysteresis should appear, as shown in insert of Figure 2b. However, in this measurement, the base temperature of VO_2_ nanowires is set to be 335 K, much higher than the phase transition temperature during the cooling process. Therefore, the jump in resistance of VO_2_ was not observed during the cooling process, which suggests that nanowires do not undergo MIT during the cooling process.

**Figure 3:**
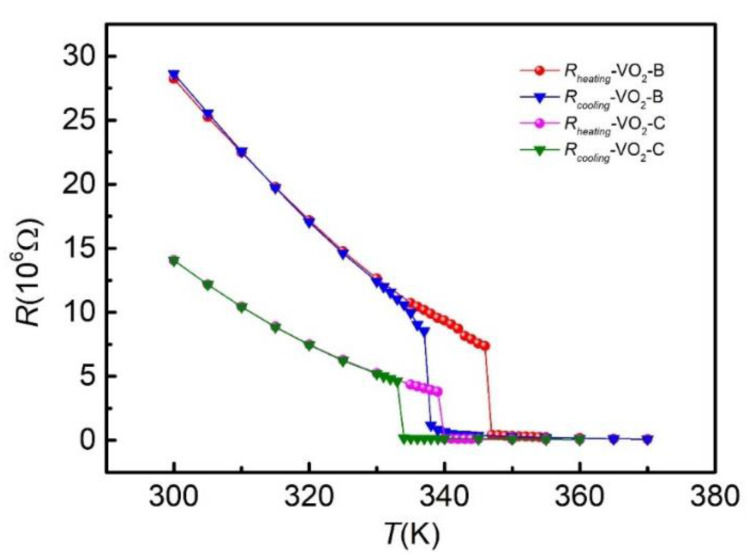
The electrical resistance as a function of temperature for VO_2_-B and VO_2_-C, respectively.

To further explore the characteristics of MIT, the electrical resistance of VO_2_ nanowires was measured by the four-electrode method from 300 K to 370 K. Figure 3 shows the temperature dependence of the electrical resistance during the cooling and heating process across the MIT. Consistent with the thermal conductance, the resistance exhibited a sharp jump across the MIT, which was over an order of magnitude. It is obvious that VO_2_ nanowires present the I phase at low temperature. However, it is pity to show that electrical resistance still decreased with increasing temperature at high temperature, similar to that in the I phase [30]. This was probably due to an incomplete MIT [31,32], where metal and insulator domains coexist in a single VO_2_ nanowire. In addition, the resistance apparently displayed a hysteresis during the temperature cycle, which is a prototypical signature of first-order MIT [33]. Notably, the hysteresis temperatures were 10 K and 7 K during the heating and cooling half cycle for VO_2_-B and VO_2_-C, respectively, which is consistent with the previous study [14]. The MIT temperatures were 347 K and 340 K for VO_2_-B and VO_2_-C, respectively, different from the result (341 K) previously reported, which may have arisen from the preparation of the sample. The four electrodes at both ends of the nanowire were deposited with Pt metal, which probably introduced a small amount of stress, resulting in the change of phase transition temperature of the VO_2_ nanowire [16,31].

**Figure 4:**
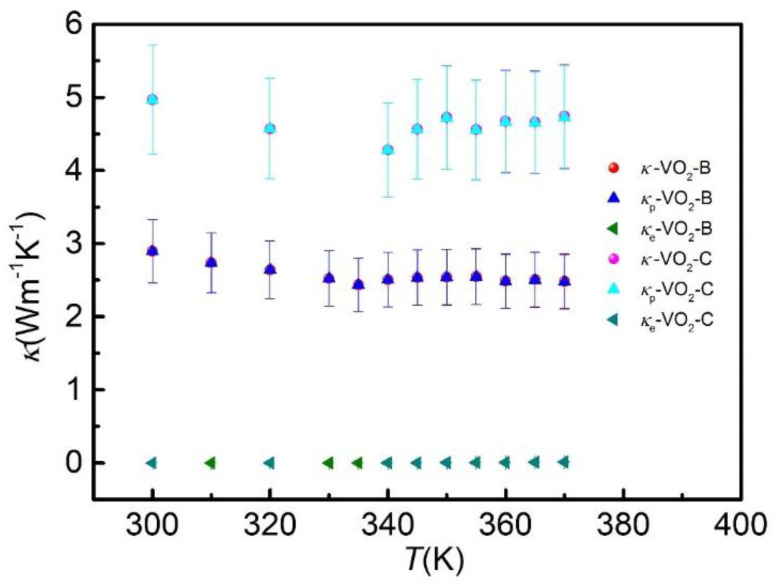
The total thermal conductivity (*κ*, red circles and magenta circles), phonon thermal conductivity (*κ_p_*, blue triangles and cyan triangles) and electronic thermal conductivity (*κ_e_*, olive triangles and dark cyan triangles) of the VO_2_ nanowire samples as a function of temperature for VO_2_-B and VO_2_-C, respectively.

To further analyze the underlying mechanisms, we studied the contribution of electronic thermal conductivity using the Wiedemann−Franz law: $\kappa_{e}=\sigma LT$, where *κ_e_*, *σ*, *L* and *T* are electronic thermal conductivity, electronic conductivity, Lorenz number and temperature of samples, respectively. Figure 4 shows the thermal conductivity as a function of temperature, where *κ_p_* and *κ* were phonon thermal conductivity and measured total thermal conductivity, respectively. *κ_p_* is determined by subtracting the *κ_e_* from *κ*. It is evident that *κ_e_* increases as the temperature rises over the entire temperature range. Remarkably, *κ_e_* increased by two orders of magnitude near the phase transition point. However, the contribution of *κ_e_* to *κ* was negligible, not exceeding 4%, no matter whether Wiedemann−Franz law was valid or not, as proposed previously [34]. *κ_p_* decreased with the increasing temperature under the phase transition temperature. Above the phase transition temperature, the trend of temperature-dependence on thermal conductivity was reversed, i.e., *κ_p_* gradually increased. The trend of *κ_p_* was consistent with that of *κ*, indicating that the reason behind the jump of *κ* mainly comes from the contribution of phonons.

The measured *η* was only 4.12% and 5.68% for VO_2_-B and VO_2_-C, respectively. It was much smaller than that in the VO_2_ films which was up to 50% [10,12]. At higher temperature, i.e., in the metal state, the electrons played a dominate role in thermal conductivity, while *κ_e_* was fully suppressed at insulating state [12]. This is the main reason to observe a great change in VO_2_ films. However, when VO_2_ films shrink to VO_2_ nanowires, both phonons and electrons are scattered by boundaries while electrons are much more severely scattered [35,36], because the electronic wave length is much larger than that in phonons, making *κ_p_* dominate thermal conductivity even in the metal state. In our work, *κ_tot_* was dominated by phonons and the contribution of the electrons was negligible in both the metal state and the insulating state. The *η* is consistent with a previous study in VO_2_ nanowires [11].

In low-dimensional materials, phonons are susceptible to boundary scattering because the size of the sample and the mean free path of phonons can be of the same order of magnitude [37,38]. In this work, the length of VO_2_-B and VO_2_-C was nearly the same, but with different diameters. The thermal conductivity of VO_2_-C was significantly higher than that of VO_2_-B, more than 70%. Therefore, the size effect may exist in VO_2_ nanowires. Similar behavior was also observed in other one-dimensional nanowires, such as silicon nanowires and zinc oxide nanowires [25,39,40,41], partially due to surface scattering and diameter-limited scattering. In addition, phonon scattering can be effectively improved by manipulating the structure of the nanomaterials such as nanoparticles [42,43], nanodots [44] and nanocomposite systems [45,46,47,48], which can lead to a series of interesting phonon transport phenomena. However, due to the limited number of samples, this result needs to be further studied.

## 3. Conclusions

In summary, the thermal conductance of VO_2_ nanowires was investigated in the temperature range of 15–370 K using the thermal bridge method. Through the phase transition from the insulator phase to metal phase, the thermal conductivity of the VO_2_ nanowire increased from 2.45 Wm^−1^K^−1^ to 2.55 Wm^−1^K^−1^, increasing by as much as 4.12%. Moreover, we found a size-dependent thermal conductivity in VO_2_ nanowires, where a thicker sample exhibited a higher thermal conductivity.

## Figures and Tables

**Table 1:** Parameters of the VO_2_ nanowires used for the thermal conductance. The lengths and diameters of the VO_2_ nanowires were measured by SEM.

Sample	Length (µm)	Diameter (nm)	*κ* (Wm^−1^K^−1^) @RT	*η* Across MIT
VO_2_-A	11.39	398.1	2.32 ± 0.12	-
VO_2_-B	20.87	530.7	3.06 ± 0.15	4.12%
VO_2_-C	20.72	708.4	4.96 ± 0.25	5.68%

## Data Availability

The data presented in this study are available on request from the corresponding author.
